# Dietary habits are associated with the prevalence of type 2 diabetes: a study among a middle eastern population

**DOI:** 10.1017/jns.2022.56

**Published:** 2022-09-19

**Authors:** Sajedeh Mahdi, Mohsen Mazidi, Ian G. Davies, Sara Beigrezaei, Hassan Mozaffari-Khosravi, Masoud Mirzaei, Katie E. Lane, Sayyed Saeid Khayyatzadeh

**Affiliations:** 1 Nutrition and Food Security Research Center, Shahid Sadoughi University of Medical Sciences, Shohadaye Gomnam Blvd, ALEM Square, Yazd, Iran; 2 Department of Nutrition, School of Public Health, Shahid Sadoughi University of Medical Sciences, Yazd, Iran; 3 Department of Twin Research, King's College London, London, UK; 4 Medical Research Council Population Health Research Unit, University of Oxford, Oxford, UK; 5 Clinical Trial Service Unit and Epidemiological Studies Unit (CTSU), Nuffield Department of Population Health, University of Oxford, Oxford, UK; 6 Research Institute of Sport and Exercise Sciences, Faculty of Science, Liverpool John Moores University, Liverpool L3 3AF, UK; 7 Yazd Cardiovascular Research Centre, Shahid Sadoughi University of Medical Sciences, Yazd, Iran

**Keywords:** Dietary habits, Fried foods, Meal frequency, Type 2 diabetes

## Abstract

Worldwide type 2 diabetes (T2D) prevalence is increasing dramatically. The present study aimed to evaluate the association between dietary habits and T2D in an Iranian adult population using a cross-sectional analysis of the Shahedieh cohort study. Participants were adults aged 35–70 years (*n* 9261) from Zarch and Shahedieh, Yazd, Iran, who attended the baseline phase of the Shahedieh cohort study. Dietary habits including meal frequency, fried-food consumption, adding salt to prepared meals and grilled-food consumption were assessed by a standard questionnaire. T2D was defined as fasting plasma glucose (FPG) ≥126 mg/dl according to the American Diabetes Association. Multiple logistic regression assessed the association between dietary habits and T2D. Individuals who consumed a meal more than six times per day compared to three times per day had greater odds for T2D (OR 2⋅503, 95 % CI 1⋅651, 3⋅793). These associations remained significant in a fully adjusted model. There was a significant association between greater intakes of fried foods and prevalence of T2D (OR 1⋅294, 95 % CI 1⋅004, 1⋅668) in the adjusted model. No significant associations were observed between other dietary habits (adding salt to prepared meals and grilled-food consumption) and odds of T2D in all crude and adjusted models. In conclusion, we have highlighted the association between meal and fried-food consumption frequencies with risk of T2D. Large longitudinal studies in different ethnicities are needed to confirm these associations.

## Introduction

While communicable diseases have largely abated over recent decades, Iran, an in transition country^(1)^ (population of about 83 million in 2019^(2)^) is now seeing a switch towards increased prevalence of non-communicable diseases (NCDs) especially type 2 diabetes (T2D). T2D has become a major public health concern in Iran with prevalence increasing from 8⋅7 % of the total population in 2000 to 11⋅3 % in 2015^(3)^. Due to increases in morbidity and mortality linked to T2D^(4)^ extensive control and preventative actions are warranted^(5)^.

Previous studies have suggested interventions to change dietary habits play an important role in ameliorating T2D prevalence and risk factors. Lotfaliany *et al.*^(6)^ showed a community-based lifestyle intervention to improve diet, increase physical activity and encourage smoking cessation prevented T2D by 30 % over 3⋅5 years in 3465 participants. Khalili-Moghadam *et al.*^(7)^ showed higher intakes of foods associated with a Mediterranean dietary pattern including fish/sea foods, legumes, nuts and monounsaturated fatty acids were associated with a decreased risk of T2D in their longitudinal study with 2139 Iranian participants. A systematic review and meta-analysis of behaviour interventions designed to encourage patients to modify their eating and activity habits, and T2D management showed behaviour interventions improved T2D self-management in 23 studies with 2208 Iranian participants^(8)^.

In consideration to dietary habits, there is mixed evidence relating to meal consumption frequency and T2D risk with differences in risk for males and females. Mekary *et al.*^(9)^ showed low eating frequency (1–2 meals/d) increased the odds of T2D (RR 1⋅25; 95 % CI 1⋅08, 1⋅45) in a 16-year follow-up cohort study of 29 206 US men, free of T2D, cardiovascular disease and cancer at baseline. In contrast, Mekary *et al.*^(10)^ showed both high- and low-frequency eating patterns were linked to T2D in 46 289 US women as part of the Nurses’ Health Study. Wang *et al.*^(11)^ showed eating 4 meals/d, compared to three meals was associated with lower risk of developing T2D (RR 0⋅76; 95 % CI 0⋅60, 0⋅97) in a Chinese population of 8874 adults over 45. In addition to meal frequency, unhealthy dietary habits such as high salt and fried food intakes have been shown to impact risk of hypertension, overweight and obesity^(12,13)^ alongside T2D. Cahill *et al.*^(14)^ showed that frequent fried-food consumption was significantly (*P* < 0⋅001) associated with risk of incident T2D in females (*n* 70 842) from the Nurses’ Health Study and males (*n* 40 789) from the Health Professionals Follow-Up Study. Farhadnejad *et al.*^(15)^ showed that adherence to healthy lifestyle score is associated with a decreased risk of type 2 diabetes in 3859 Iranian adults. Increased sodium intake was shown to significantly (*P* < 0⋅05) decrease healthy lifestyle scores across the four quartiles. Further research is needed to evaluate the combined associations between meal frequency^(11)^, high salt intakes^(16)^, frequency of fried/grilled-food consumption^(13)^ with the prevalence of T2D.

There is a gap in the evidence for observational studies that evaluate dietary habits and T2D prevalence in Iran. Further evaluation of dietary habits is needed to inform future randomised controlled trials aiming to ameliorate T2D prevalence; therefore, the aim of the present study was to examine associations between dietary habits and T2D in a large population of adults from the Shahedieh cohort study.

## Methods and materials

### Study population and data collection

The present study is a cross-sectional analysis of the Shahedieh cohort study, a part of the PERSIAN multi-centre cohort study conducted in a representative sample of the Iranian adult population aged 35–70 years old^(17)^. The Shahedieh cohort study recruited around 10 000 adults aged over 35 years living in two municipal areas of Yazd city (Zarch and Shahedieh), Yazd province, Iran. Details of the PERSIAN cohort study protocol are provided elsewhere^(18)^. Briefly, the participants were selected by a multistage cluster random sampling method after giving written informed consent. The eligible participants were then invited to give blood samples and provide data on general characteristics, demographic, dietary intake, smoking and other lifestyle-related data. Anthropometric and blood pressure measurements were also conducted for all attendants. All data were collected by trained interviewers^(18)^. Data on 9975 adults were provided. Medical history was assessed by a self-reported questionnaire, participants with a history of cancer (skin, breast, stomach, colorectal, bladder, blood, oesophageal, prostate, lung, central nervous system, larynx, tongue, cervix/uterine, ovary) or autoimmune diseases (rheumatoid disorders, multiple sclerosis) were excluded. Following exclusions, 9261 participants remained for the present analysis. This study was conducted according to the guidelines laid down in the Declaration of Helsinki and all procedures involving research study participants were approved by the ethics committee of Shahid Sadoughi University of Medical Sciences (approval code: IR.SSU.SPH.REC.1397.161). Written informed consent was obtained from all subjects/patients.

### Dietary intakes and habits assessments

The study participants were interviewed by trained nutritionists who completed a semi-quantitative food frequency questionnaire (FFQ) with 121 items asking about their dietary intakes over the past year. Participants were asked two questions about each food item: (1) the frequency consumption (number of times per month, week or day the food was consumed) in the previous year and (2) the amount of food usually consumed each time (portion size based on the standard serving sizes commonly consumed by Iranians^(19)^). A ‘meal’ was defined as a mixed dish combining various ingredients prepared by different methods of cooking, consumed either at home or in a restaurant excluding snacks such as fruit, sweets and crisps^(19)^. All reported intakes were converted to g/d by using household portion sizes of consumed foods. The USDA food database was used to calculate nutrient intakes^(20)^.

Dietary habits including meal frequency (<3 meals/d, 3 meals/d, 4–6 meals/d and >6 meals/d), fried-food consumption (<1 time/month, 1–3 times/month, 1–3 times/week and daily), adding salt to prepared meal (no, sometimes, yes), grilled-food consumption (<1 time/month, 1–3 times/month and 3 times/month) were collected through a dietary habit questionnaire^(19)^.

### Anthropometric measurement

Anthropometric parameters (weight, height) were measured by a trained investigator. Weight was measured while the participants were with minimum clothing and without shoes using a digital scale (SECA, model 755, Germany). Participants’ height was measured by a tape measure attached to a flat wall with a precision of 0⋅5 cm. Body mass index (BMI) was calculated by dividing weight (kg) by height (metres) squared.

### Physical activity measurement

Participants were asked about their usual physical activity levels in the last year and if they had seasonal jobs using a questionnaire previously validated in an Iranian population^(21)^. The information gathered in the questionnaire was converted to the metabolic equivalent of task hours per week (MET-h/wk)^(22)^ and then categorised to sedentary, moderate and active based on the median of MET-h/wk levels.

### Biochemical assessment

Participants were asked to abstain from food on the test day and provide 25 ml of fasted blood using Vacutainers (Greiner Bio-One International GmbH, Kremsmunster, Austria). The blood was centrifuged and fractioned into various aliquots, which were labelled and stored in freezers (−70°C). In addition to the stored samples, a small amount of blood was used to measure fasting blood glucose (FBG) using the enzymatic colorimetric method. The commercially available enzymatic reagents (Pars Azmoon, Tehran, Iran), adapted to an auto analyzer system (Selectra E; Vitalab, Holliston, the Netherlands), were applied for all measurements.

### Diagnosis of type 2 diabetes

The following criteria were used to determine whether a participant had T2D and the prevalence was calculated accordingly. History of T2D was recorded by practitioner diagnosis over a lifetime according to patients’ interviews. T2D was determined as fasting plasma glucose (FPG) ≥126 mg/dl (7⋅0 mmol/l) as defined by American Diabetes Association^(23)^ (ADA). Any participant with FBG ≥126 mg/dl or receiving blood glucose lowering drugs was classified as having T2D.

### Statistical analysis

Comparisons of quantitative (age, physical activity, BMI, waist circumference (WC), fasting blood sugar (FBS) and dietary intakes) and qualitative data (gender, smoking, cardiovascular or liver disease presence) across categories of dietary habits were assessed using One-way ANOVA and *χ*^2^ tests, respectively. Bonferroni *post hoc* analysis was applied *post hoc* tests within the ANOVA tests. To find the association between dietary habits and T2D, we used logistic regression in different models. Initially, the associations were adjusted for age and total energy intake. Further adjustments were done for physical activity, BMI, gender and smoking in Model II. The history of cardiovascular diseases and liver diseases (accounting for: hypertension, ischaemic heart disease (angina, heart failure), cerebrovascular accident, fatty liver (diagnosed by Dr), hepatitis B, hepatitis C were adjusted in Model III. Final adjustments were performed for wealth score index (WSI) score. All statistical analyses were done using the Statistical Package for Social Sciences (SPSS, version 15.0 for Windows, 2006, SPSS, Inc, Chicago, IL). *P*-values less than 0⋅05 were considered statistically significant.

## Results

Out of the total participants, a sample of *n* 9261 with mean age 48⋅3 ± 9⋅6 (51⋅2 % male) was eligible for current analysis with T2D prevalence of 20⋅8 % (*n* 2962). The general characteristics of study participants according to the subgroups of dietary habits are provided in Table 1. There were significant differences for age, gender, physical activity, BMI and cardiovascular disease between categories of all dietary habits except ‘meal frequency’. Individuals with the highest meal frequency exhibited higher FBS but lower smoking habits. Individuals with the lowest fried-food consumption had significantly higher age, BMI, WC, FBS, CVD and liver diseases, and lower physical activity. For those who did not add salt to meals, age, BMI, WC, FBS, CVD were higher whereas PA and smoking were lower than in those who added salt. The highest prevalence of liver diseases was found in subjects in the lowest category of ‘fried-food consumption’. We did not find any significant differences in the distribution of other variables in dietary habits categories.

**Table 1.** Characteristics of study participants according to dietary habits categories

		Age	Gender (% Male)	Physical activity (MET-h/wk)	BMI (kg/m²)	WC (cm)	FBS (mg/dl)	Smoking (%)	Cardiovascular diseases (%)	Liver diseases (%)
Meal frequency	<3 meals/d	48⋅39 ± 9⋅87	182(49⋅1)	41⋅23 ± 8⋅04	28⋅24 ± 5⋅55	95⋅18 ± 13⋅24	105⋅128 ± 37⋅39^ab^	51⋅97	23⋅9	13⋅9
	3 meals/d	48⋅82 ± 10⋅11	801(53⋅3)	41⋅23 ± 7⋅78	28⋅29 ± 5⋅21	95⋅68 ± 12⋅27	107⋅69 ± 42⋅96^ab^	44⋅65	25⋅2	14⋅7
	4–6 meals/d	48⋅16 ± 9⋅46	3535(50⋅7)	41⋅03 ± 7⋅10	28⋅41 ± 4⋅71	96⋅12 ± 11⋅26	107⋅24 ± 40⋅47^a^	38⋅33	24⋅1	15⋅5
	>6 meals/d	48⋅44 ± 9⋅61	144(54⋅8)	41⋅86 ± 7⋅62	28⋅41 ± 4⋅68	96⋅80 ± 11⋅40	114⋅197 ± 51⋅42^b^	37⋅07	26	15⋅5
	*P*-value	0⋅109	0⋅155	0⋅239	0⋅781	0⋅172	0⋅039	<0⋅001	0⋅725	0⋅752
Fried-food consumption	<1 time/month	54⋅41 ± 9⋅65^a*^	247(43⋅3)	40⋅22 ± 7⋅66^a^	28⋅86 ± 5⋅08^a^	97⋅66 ± 11⋅88^a^	112⋅83 ± 42⋅85^a^	35⋅39	43	19⋅2
	1–3 times/month	49⋅26 ± 9⋅76^b^	1322(49⋅6)	40⋅91 ± 7⋅49^ab^	28⋅47 ± 4⋅80^ab^	96⋅64 ± 11⋅38^ab^	107⋅98 ± 41⋅15^ab^	45⋅86	26⋅4	17⋅4
	>3 times/month	47⋅17 ± 9⋅26^cd^	2550(53⋅8)	41⋅24 ± 7⋅22^b^	28⋅25 ± 4⋅80^b^	95⋅51 ± 11⋅50^c^	106⋅02 ± 39⋅41^b^	39⋅87	21⋅6	14⋅2
	Daily	47⋅56 ± 9⋅15^cd^	543(48⋅1)	41⋅33 ± 6⋅70^b^	28⋅50 ± 4⋅90^ab^	95⋅96 ± 11⋅63^bc^	109⋅30 ± 46⋅64	33⋅05	21⋅6	13⋅3
	*P*-value	<0⋅001	<0⋅001	0⋅004	<0⋅001	<0⋅001	<0⋅001	<0⋅001	<0⋅001	<0⋅001
Adding salt to prepared meal	No	48⋅73 ± 9⋅68^a^	3365(48⋅1)	40⋅95 ± 7⋅13^a^	28⋅55 ± 4⋅82^a^	96⋅38 ± 11⋅40^a^	108⋅01 ± 41⋅63	36⋅65	27⋅3	15⋅7
	Sometimes	47⋅27 ± 9⋅26^b^	502(58⋅6)	41⋅51 ± 7⋅17^b^	28⋅14 ± 4⋅75^b^	95⋅33 ± 11⋅53^b^	105⋅60 ± 38⋅93	41⋅81	16	14
	Yes	46⋅50 ± 9⋅06^b^	795(63⋅2)	41⋅58 ± 8⋅07	27⋅62 ± 4⋅89^c^	94⋅54 ± 12⋅09^b^	105⋅44 ± 39⋅77	51⋅82	13⋅8	13⋅9
	*P*-value	<0⋅001	<0⋅001	0⋅004	<0⋅001	<0⋅001	0⋅051	<0⋅001	<0⋅001	0⋅133
Grilled-food consumption	<1 time/month	50⋅49 ± 10⋅03^a^	1450(43⋅5)	40⋅82 ± 7⋅15^a^	28⋅61 ± 4⋅99^a^	96⋅40 ± 11⋅90	109⋅54 ± 43⋅14^a^	32⋅56	29⋅9	14⋅7
	1–3 times/month	47⋅03 ± 8⋅98^b^	2308(53⋅6)	41⋅34 ± 7⋅35^b^	28⋅25 ± 4⋅76^b^	95⋅82 ± 11⋅35	105⋅66 ± 38⋅80^b^	43⋅30	20⋅6	15⋅8
	>3 times/month	46⋅95 ± 9⋅43^b^	904(61⋅4)	40⋅98 ± 7⋅30^ab^	28⋅26 ± 4⋅66^ab^	95⋅82 ± 11⋅15	107⋅80 ± 42⋅88^ab^	47⋅36	22⋅5	15⋅4
	*P*-value	<0⋅001	<0⋅001	0⋅007	0⋅003	0⋅067	<0⋅001	<0⋅001	<0⋅001	0⋅384

* a,b,c,d: the results of Bonferroni test. Data was provided by mean±SD for continuous and n (%) for categorical variables.

Dietary nutrient intakes (adjusted) of study population among dietary habits categories are shown in Table 2. The participants in the highest category of all dietary habits had higher amounts of energy, protein, fat, carbohydrate, sucrose, fibre, vitamin C, beta-carotene, folate, potassium and sodium. After adjustment for energy, significant differences disappeared only for fat or carbohydrate intake among categories of ‘adding salt to meal’, folate among categories of ‘fried-food consumption’ and sucrose among ‘grilled-food consumption’. Crude unadjusted dietary nutrient intakes are shown in Supplementary Material S1.

**Table 2.** Comparison of energy-adjusted* nutrient intake according to categories of dietary habits

		Energy intake (kcal)	Protein (g/d)	Fat (g/d)	Carbohydrate (g/d)	Sucrose (g/d)	Fibre total (g/d)	Vitamin c (mg/d)	Beta-Carotene (μg/d)	Folate (μg/d)	Potassium (mg/g)	Sodium (mg/g)
Meal frequency	<3 meals/d	2497⋅47 ± 1224⋅33^a**^	25⋅51 ± 4⋅82^a^	28⋅09 ± 9⋅26^ab^	164⋅16 ± 21⋅67	19⋅39 ± 17⋅18^a^	8⋅34 ± 2⋅84^a^	40⋅99 ± 29⋅22^a^	1289⋅25 ± 1470⋅39^a^	137⋅69 ± 57⋅13^a^	1199⋅75 ± 406⋅33^a^	1582⋅18 ± 572⋅34^a^
	3 meals/d	2690⋅67 ± 1172⋅56^b^	26⋅73 ± 4⋅19^b^	27⋅82 ± 8⋅16^a^	163⋅51 ± 17±96	15⋅29 ± 10⋅3^b^	8⋅79 ± 2⋅58^b^	43⋅76 ± 29⋅75^a^	1397⋅68 ± 1355⋅04^a^	137⋅61 ± 49⋅47^ab^	1198⋅31 ± 376⋅52^a^	1584 ± 548⋅29^ab^
	4–6 meals/d	2919⋅79 ± 1136⋅28^c^	26⋅92 ± 3⋅55^b^	28⋅68 ± 7⋅51^b^	162⋅29 ± 16⋅45	14⋅56 ± 8⋅48^c^	9⋅7 ± 2⋅6^c^	52⋅92 ± 31⋅73^b^	1712⋅93 ± 1415⋅74^b^	142⋅09 ± 46⋅59^ac^	1277⋅62 ± 357⋅05^b^	1523⋅57 ± 457⋅15^ac^
	>6 meals/d	3482⋅46 ± 1378⋅54^d^	26⋅67 ± 3⋅8^b^	28⋅4 ± 7⋅08^ab^	164⋅55 ± 16⋅06	15⋅94 ± 9⋅04^cb^	10⋅75 ± 2⋅84^d^	68⋅35 ± 40⋅2^c^	2096⋅9 ± 1787⋅95^c^	156⋅44 ± 52⋅68^d^	1426⋅09 ± 371⋅86^c^	1484⋅04 ± 497⋅77^acd^
	*P*-value	<0⋅001	<0⋅001	0⋅001	0⋅004	<0⋅001	<0⋅001	<0⋅001	<0⋅001	<0⋅001	<0⋅001	<0⋅001
Fried-food consumption	<1 time/month	2269⋅83 ± 1015⋅44^a^	27⋅62 ± 4⋅32^a^	26⋅37 ± 8⋅02^a^	166⋅75 ± 17⋅74^a^	13⋅83 ± 8⋅46^a^	9⋅89 ± 3⋅34^a^	50⋅06 ± 32⋅95^abc^	1566⋅23 ± 155a4⋅96^abc^	142⋅23 ± 52⋅54^a^	1313⋅22 ± 440⋅33^a^	1607⋅91 ± 625⋅54
	1–3 times/month	2711⋅41 ± 1065⋅11^b^	26⋅69 ± 3⋅76^b^	27⋅88 ± 7⋅41^b^	164⋅19 ± 16⋅63^b^	14⋅82 ± 10⋅02^ab^	9⋅55 ± 2⋅72^b^	50 ± 30⋅57^ab^	1659⋅77 ± 1504⋅06^abc^	139⋅8 ± 47⋅96^b^	1267⋅44 ± 366⋅54^b^	1520⋅22 ± 461⋅05
	>3 times/month	2992⋅82 ± 1184⋅94^c^	26⋅87 ± 3⋅64^b^	28⋅88 ± 7⋅68^c^	161⋅74 ± 16⋅89^c^	15⋅07 ± 9⋅24^ab^	9⋅49 ± 2⋅53^b^	51⋅87 ± 32⋅32^abc^	1685⋅19 ± 1375⋅24^ab^	141⋅95 ± 47⋅24^b^	1260⋅96 ± 356⋅04^b^	1534⋅82 ± 482⋅48
	Daily	3120⋅08 ± 1163⋅63^d^	26⋅58 ± 3⋅72^b^	29⋅5 ± 7⋅92^c^	160⋅59 ± 16⋅87^c^	15⋅07 ± 8⋅75	9⋅46 ± 2⋅59^b^	53⋅19 ± 33⋅05^abc^	1560⋅01 ± 1395⋅81^abc^	143⋅96 ± 47⋅34^b^	1257⋅36 ± 357⋅55^b^	1532⋅29 ± 425⋅29
	*P*-value	<0⋅001	<0⋅001	<0⋅001	<0⋅001	0⋅023	0⋅007	0⋅013	0⋅025	0⋅074	0⋅011	0⋅001
Adding salt to prepared meal	No	2790⋅07 ± 1118⋅14^a^	26⋅93 ± 3⋅75^a^	28⋅53 ± 7⋅69	162⋅59 ± 16⋅98	14⋅54 ± 9⋅02^a^	9⋅68 ± 2⋅7^a^	52⋅39 ± 32⋅46^a^	1696⋅97 ± 1458⋅06^a^	142⋅8 ± 48⋅51^a^	1282⋅51 ± 369⋅75^a^	1506⋅07 ± 470⋅15^a^
	Sometimes	3027⋅97 ± 1202⋅95^b^	26⋅81 ± 3⋅45^a^	28⋅16 ± 7⋅75	162⋅92 ± 16⋅75	15⋅03 ± 8⋅87^a^	9⋅12 ± 2⋅36^b^	48⋅93 ± 31⋅15^b^	1484⋅9 ± 1189⋅02^b^	138⋅56 ± 42⋅7^b^	1213⋅81 ± 336⋅65^b^	1563⋅76 ± 440⋅05^b^
	Yes	3285⋅96 ± 1282⋅24^c^	26⋅24 ± 3⋅79^b^	28⋅62 ± 7⋅64	162⋅63 ± 16⋅9	16⋅91 ± 11⋅23^b^	8⋅95 ± 2⋅47^b^	47⋅35 ± 29⋅25^b^	1536⋅35 ± 1398⋅67^b^	136⋅9 ± 46⋅89^b^	1207⋅44 ± 349⋅45^b^	1674⋅2 ± 533⋅26^c^
	*P*-value	<0⋅001	<0⋅001	0⋅362	0⋅870	<0⋅001	<0⋅001	<0⋅001	<0⋅001	<0⋅001	<0⋅001	<0⋅001
Grilled-food consumption	<1 time/month	2606⋅05 ± 1109⋅49^a^	26⋅83 ± 3⋅73^ab^	27⋅61 ± 8⋅19^a^	164⋅41 ± 17⋅77^a^	14⋅78 ± 9⋅75	9⋅5 ± 2⋅74^ab^	48⋅97 ± 32⋅12^a^	1451⋅79 ± 1295⋅55^a^	141⋅04 ± 47⋅9^a^	1248⋅32 ± 372⋅55^a^	1585⋅34 ± 475⋅31^a^
	1–3 times/month	2967⋅53 ± 1128⋅59^b^	26⋅75 ± 3⋅62^a^	28⋅82 ± 7⋅37^b^	162⋅01 ± 16⋅35^b^	14⋅86 ± 9⋅18	9⋅49 ± 2⋅56^a^	51⋅28 ± 29⋅54^b^	1709⋅21 ± 1422⋅87^b^	140⋅57 ± 46⋅43^a^	1260⋅97 ± 352⋅96^a^	1514⋅16 ± 464⋅79^b^
	>3 times/month	3249⋅79 ± 1240⋅86^c^	27⋅04 ± 4⋅07^b^	29⋅61 ± 7⋅21^c^	160⋅41 ± 16⋅37^c^	15⋅4 ± 9⋅08	9⋅69 ± 2⋅72^b^	57⋅04 ± 37⋅31^c^	1954⋅6 ± 1649⋅53^c^	145⋅77 ± 51⋅33^b^	1318⋅58 ± 379⋅06^b^	1480⋅74 ± 523⋅54^b^
	*P*-value	<0⋅001	0⋅035	<0⋅001	<0⋅001	0⋅090	0⋅030	<0⋅001	<0⋅001	0⋅001	<0⋅001	<0⋅001

* per 1000 kcal energy intake.

** a,b,c,d: the results of Bonferroni test. Data was provided by mean±SD.

Multivariate-adjusted odds ratios for T2D across categories of dietary habits are presented in Table 3. Individuals who consumed their meal more than three times a day had greater odds for T2D (OR 2⋅503, 95 % CI 1⋅651, 3⋅793, *P* < 0⋅001) compared to those who consumed fewer than 3 meals/d. These associations remained significant after adjustment in Model II (OR 2⋅163, 95 % CI 1⋅128, 4⋅148, *P* < 0⋅026), III (OR 2⋅424, 95 % CI 1⋅577, 3⋅725, *P* < 0⋅001) and IV (OR 2⋅213, 95 % CI 1⋅140, 4⋅296, *P* < 0⋅024). There was a significant direct relationship between greater intakes of fried foods and prevalence of T2D (OR 1⋅294, 95 % CI 1⋅004, 1⋅668, *P* < 0⋅027) in the third adjusted model, but these associations were not significant in the first, second and final models.

**Table 3.** Multivariable-adjusted odds ratios (95 % CI) for type 2 diabetes mellitus according to categories of dietary habit

		Model I	Model II	Model III	Model IV
Meal frequency	<3 meals/d	Ref	Ref	Ref	*Ref*
	3 meals/d	1⋅245(0⋅901–1⋅720)	1⋅116(0⋅697–1⋅788)	1⋅205(0⋅864–1⋅680)	1⋅15(0⋅722–1⋅881)
	4–6 meals/d	1⋅631(1⋅208–2⋅203)	1⋅297(0⋅844–1⋅993)	1⋅579(1⋅160–2⋅150)	1⋅346(0⋅867–2⋅088)
	>6 meals/d	2⋅503(1⋅651–3⋅793)	2⋅163(1⋅128–4⋅148)	2⋅424(1⋅577–3⋅725)	2⋅213(1⋅140–4⋅296)
	*P*-value	<0⋅001	0⋅026	<0⋅001	0⋅024
Fried-food consumption	<1 time/month	*Ref*	*Ref*	*Ref*	*Ref*
	1–3 times/month	0⋅914(0⋅739–1⋅132)	0⋅794(0⋅544–1⋅161)	0⋅995(0⋅796–1⋅242)	0⋅838(0⋅567–1⋅237)
	1–3 times/week	0⋅931(0⋅756–1⋅145)	0⋅754(0⋅525–1⋅084)	1⋅034(0⋅833–1⋅285)	0⋅805(0⋅554–1⋅170)
	Daily	1⋅171(0⋅917–1⋅496)	0⋅950(0⋅628–1⋅437)	1⋅294(1⋅004–1⋅668)	1⋅012(0⋅661–1⋅549)
	*P*-value	0⋅135	0⋅960	0⋅027	0⋅816
Adding salt to prepared meal	No	*Ref*	*Ref*	*Ref*	*Ref*
	Sometimes	0⋅852(0⋅700–1⋅036)	0⋅905(0⋅678–1⋅207)	0⋅963(0⋅789–1⋅177)	0⋅989(0⋅739–1⋅325)
	Yes	0⋅880(0⋅744–1⋅042)	0⋅975(0⋅762–1⋅247)	1⋅045(0⋅880–1⋅242)	1⋅044(0⋅813–1⋅341)
	*P*-value	0⋅727	0⋅065	0⋅716	0⋅768
Grilled-food consumption	<1 time/month	*Ref*	*Ref*	*Ref*	*Ref*
	1–3 times/month	1⋅035(0⋅919–1⋅167)	1⋅003(0⋅820–1⋅226)	1⋅084(0⋅958–1⋅227)	1⋅048(0⋅852–1⋅290)
	>3 times/month	1⋅054(0⋅893–1⋅244)	0⋅911(0⋅693–1⋅197)	1⋅057(0⋅890–1⋅254)	0⋅915(0⋅690–1⋅213)
	*P*-value	0⋅580	0⋅351	0⋅485	0⋅689

Model I: Adjusted for age, total energy intake.

Model II: Additionally adjustments for physical activity, body mass index, gender and smoking.

Model III: Furthermore adjustments for history of cardiovascular diseases and liver diseases.

Model IV: Additionally adjustments for WSI score.

No significant associations were observed between other dietary habits (adding salt to prepared meal and grilled-food consumption) and odds of T2D in all models.

## Discussion

The increasing prevalence of T2D represents a major present and future public health challenge^(24,25)^. Modification of dietary habits in addition to dietary intakes is important and should be considered as a public health strategy to tackle T2D^(26)^. The present study evaluated dietary habits including meal frequency, fried-food consumption, addition of salt to prepared meals and grilled-food consumption and the risk of T2D in 9261 participants of the Shahedieh cohort study. The present study highlighted associations between the dietary habits of participants in a cross-sectional analysis of the Shahedieh cohort study. Individuals who consumed a meal more than six times per day had greater odds for T2D compared to those with lower-frequency meal consumption. These associations remained significant in a fully adjusted model. There was also a significant direct relationship between greater intakes of fried foods and prevalence of T2D after adjusting for history of CVDs and liver disease.

The participants who consumed >6 meals/d had significantly higher total energy intakes. Protein, fat, carbohydrate, sugar, fibre and micronutrient intakes were also significantly increased in participants who consumed >6 meals/d, which could indicate minimal consumption of energy-dense nutrient poor foods. There were no significant differences for age, gender, physical activity, BMI, WC or FBS for meal frequency, however participants who consumed >6 meals/d were significantly less likely to smoke and significantly more likely to have greater odds for T2D. Low meal frequency interventions have previously been shown to ameliorate T2D markers in comparison to consumption of a higher frequency of smaller meals a day in combination with hypocaloric diets^(27,28)^. Eating a larger breakfast and lunch was shown to be more effective than six smaller meals during the day^(27,28)^. Coupled with previous evidence, our findings show decreased odds for T2D with lower meal frequency and total energy consumption; however, meal timings were not established in our study. Indeed, very few studies have focused on meal timing and T2D and there is a lack of consensus on the definition of a meal, snack and meal timing in this research field^(29)^. The present study shares this lack of consensus; despite having a definition to represent a ‘meal’, the consumption frequency options available in the questionnaire were ambiguous with some options including snacks and some that did not^(19)^.

Our findings show the habit of adding salt to a prepared meal corresponded to significantly higher sodium intake. Predictably those who stated they did not add salt to meals had significantly lower sodium intakes. Smokers were significantly more likely to add salt to prepared meals. Consumption of salt among the Iranian population is higher than the level of 5 g/d recommended by the WHO^(30,31)^. The mean salt intake for a cohort of 18 260 Iranian adults over the age of 25 was 9⋅52 g/d and 41⋅2 % consumed twice the recommended amount of salt^(31)^. Sodium intakes were also significantly higher in the groups who consumed >6 meals/d and fried food daily.

Daily consumers of fried food were not in the highest BMI category, which is in agreement with findings by Saneei *et al.*^(26)^ who showed moderate-to-high intake of fatty foods was inversely associated with central obesity in 7958 Iranian adults. These surprising findings may be linked to the fact that Iranians obtain more than 60 % of their calories from carbohydrates, particularly refined grains with high glycemic index and glycemic load. Refined carbohydrate consumption in Iran is among the highest levels in the world^(26,32,33)^. However, daily fried-food consumption gave significantly increased odds for T2D in the present study where adjustments were made for history of CVD and liver disease. Despite our participants being younger and more physical active, fried foods appear to be associated with diabetes. However, high energy intake is clearly a confounding variable that attenuates this association, as does a higher number of more physically active younger females with lower BMI. Therefore, being younger, female, having lower BMI, energy intake and increased physically activity is protective of higher consumption of fried food. But once CVD and liver disease is factored into the model, the association with T2D is significant. Meaning the profile of those with T2D are older, male, higher BMI, higher CVD and higher liver disease. Therefore, CVD and liver disease are driving the significant association of fried food with T2D. In other words, CVD and liver disease are co-morbidities to T2D observed with daily fried-food consumption.

The evidence on the relationships between fried food and T2D is mixed. A meta-analysis of large prospective studies investigating Western dietary patterns (DP), showed a positive relationship with T2D^(34,35)^, with up to 41 % increased odds of T2D with a Western DP compared to a prudent/healthier DP^(35)^. However, direct data on fried foods is less abundant, a pooled analysis of two large prospective cohorts showed relative risk of T2D increased progressively with the frequency of fried food intake from 1 to 7 d/week even after adjustment of a number of covariates^(14)^. This is in agreement with other studies and systematic reviews^(36–38)^, but others have found non-significant or no relationship with certain fried food and T2D^(13,39,40)^. Moreover, a meta-analysis by Qin *et al.*^(13)^ showed after subgroup analysis, significant associations were found for studies conducted in the USA and Asia with larger sample sizes and a focus on total fried food. The latter agrees with the present study, and highlights the limitations of other studies that only focused on individual fried foods (i.e. fish and potatoes)^(39,40)^. For the present study, the FFQ was designed to get an overview of fried-food consumption frequency while effectively collecting data from a large sample size. The questionnaire did not establish different types of fried foods (potatoes, meat, vegetables, etc.) nor the quality or quantity of dietary fats used during frying, representing a limitation that should be addressed in future studies.

It is important to consider the type of oil and cooking practices. Frying increases the calorie content and palatability of a food potentially leading to weight gain, but also alters the chemical nature of the specific oils used^(41)^. With high temperatures, some fatty acids convert to *trans*-fatty acids (TFA), and other potentially harmful bioactive compounds. TFA are well-known risk factors for T2D and other cardiometabolic disease, and are relatively high in some fried foods such as kebabs^(42,43)^ a popular food choice in Iran^(44)^.

Several limitations need attention. First, this was a cross-sectional study; therefore, it is fundamentally difficult to determine whether or not the observed relationships are causal. Second, data provided on dietary habits used self-reported questionnaires. While we have adjusted for energy intake, adjustment for macro and micronutrient intake falls outside the remit of this study. It should also be considered that the study participants were selected from semi-urban areas of the Yazd city and the generalisation of the findings should be done with caution. In addition, although we tried to adjust the maximum number of possible confounding variables, residual confounding from unknown or unmeasured confounders is inevitable. On the other hand, the present study benefited from a large sample size, which is a key strength.

In conclusion, our study highlighted associations between the dietary habits of participants in a population-based cohort and T2D. We established that individuals who consumed a meal more than 6 times per day had greater odds for T2D compared to those with a low frequency. These associations remained significant in a fully adjusted model. There was a significant direct relationship between greater intakes of fried foods and prevalence of T2D after adjusting for history of CVDs and liver disease. No significant associations were observed between other dietary habits including adding salt to prepared meals and grilled-food consumption and odds of T2D in all models. Further studies to evaluate T2D prevalence with the nutritional quality of the meals consumed and the types of fried foods in different ethnicities are warranted.

## Supplementary material

For supplementary material accompanying this paper visit https://doi.org/10.1017/jns.2022.56. click here to view supplementary material
