# Supplementary material for: Dietary habits are associated with the prevalence of type 2 diabetes: a study among a middle eastern population
Source: J Nutr Sci. 2022 Sep 19;11:e78. doi: 10.1017/jns.2022.56 (PMC9554423; doi:10.1017/jns.2022.56)
Supplement: Supplementary file 1 [file S2048679022000568sup001.docx]

**S1 –** Unadjusted comparison of daily intake of energy and nutrients according to categories of dietary habits.

| Sodium  (mg/g) | Potassium  (mg/g) | Folate  (µg/d) | Beta Carotene (µg/d) | Vitamin c (mg/d) | Fiber total (g/d) | Sucrose (g/d) | Carbohydrate (g/d) | Fat  (g/d) | Protein (g/d) | Energy intake ^I^ (Kcal/d) |  | |
| --- | --- | --- | --- | --- | --- | --- | --- | --- | --- | --- | --- | --- |
| 3746.28±1871.34 | 2933.87±2021.25 | 334.55±221.21 | 3174.10±4814.62 | 101.04±126.56 | 20.25±12.74 | 50.26±64.39 | 412.27±219.09 | 69.28±39.73 | 63.03±31.93 | 2497.47±1224.33¹ | < 3 meal/day | Meal frequency |
| 4139.56±2109.67 | 3102.92±1436.51 | 359.09±186.75 | 3629.40±4023.32 | 113.11±91.89 | 23.13±11.22 | 40.37±32.03 | 442.90±210.04 | 73.32±36.40 | 71.69±33.63 | 2690.67±1172.56 | 3 meal/day |  |
| 4354.12±1979.65 | 3631.92±1558.44 | 406.01±195.57 | 4828.87±4375.35 | 150.17±116.76 | 27.80±11.85 | 42.66±35.24 | 476.87±204.02 | 82.24±35.84 | 78.31±32.06 | 2919.79±1136.28 | 4-6 meal/day |  |
| 5020.53±2295.73 | 4835.04±2013.63 | 528.38±236.01 | 6995.97±6577.66 | 234.40±173.98 | 36.55±15.04 | 57.17±45.89 | 578.24±247.84 | 96.84±40.97 | 91.67±36.31 | 3482.46±1378.54 | > 6 meal/day |  |
| <0.001 | <0.001 | <0.001 | <0.001 | <0.001 | <0.001 | <0.001 | <0.001 | <0.001 | <0.001 | <0.001^II^ | *p* value |  |
| 3568.29 ± 2023.5 | 2890.68 ± 1486.17 | 315.11±166.94 | 3458.17±3899.83 | 108.68 ± 77.72 | 21.88 ± 10.66 | 31.07±24.84 | 380.27±180.61 | 58.86 ± 32.7 | 62.5 ±29.93 | 2269.84±1015.45 | < 1 time/month | Fried-food consumption |
| 3994.92 ± 1713.05 | 3347.75 ± 1473.89 | 369.76±178.25 | 4377.10±4389.56 | 132.81±100.97 | 25.44 ± 11.27 | 40.38±35.68 | 448.54±194.32 | 74.12 ± 31.9 | 71.88±29.06 | 2711.42±1065.11 | 1-3 time/month |  |
| 4491.97 ± 2112.52 | 3679.21 ± 1643.19 | 416.10±208.47 | 4890.16±4545.05 | 151.42±128.56 | 27.95 ± 12.45 | 45.11±39.25 | 487.36±212.56 | 84.77±37.33 | 80.22±33.89 | 2992.83±1184.95 | > 3 time/month |  |
| 4689.70 ± 2058.53 | 3825.51 ± 1672.71 | 437.67±198.43 | 4694.90±4461.67 | 161.10±117.18 | 29.10 ± 12.88 | 46.43±32.46 | 505.60±218.61 | 89.86±38.79 | 82.74±33.83 | 3120.04±1209.74 | Daily |  |
| <0.001 | <0.001 | <0.001 | <0.001 | <0.001 | <0.001 | <0.001 | <0.001 | <0.001 | <0.001 | <0.001 | *p* value |  |
| 4090.57±1890.79 | 3486.03±1561.91 | 389.94±193.78 | 4603.84±4411.43 | 143.4±119.55 | 26.54±11.77 | 40.63±34.94 | 456.34±199.99 | 78.29±35.32 | 74.83±31.6 | 2790.07±1118.14 | No | Adding salt to prepared meal |
| 4626.12±2001.94 | 3585.33±1614.84 | 409.78±185.78 | 4399.86±4028.16 | 142.85±104.84 | 27.31±12.77 | 44.85±32.67 | 497.39±220.55 | 83.35±36.88 | 80.91±33.39 | 3027.98±1202.96 | Sometimes |  |
| 5335.29±2326.11 | 3888.34±1797 | 441.19±227.91 | 4905.06±5011.45 | 151.75±115.22 | 29.2±13.83 | 54.99±46.3 | 538.52±230.57 | 92.01±40.75 | 86.27±36.57 | 3285.96±1282.24 | Yes |  |
| <0.001 | <0.001 | <0.001 | 0.026 | 0.061 | <0.001 | <0.001 | <0.001 | <0.001 | <0.001 | <0.001 | *p* value |  |
| 4022.71±1932.73 | 3142.61±1411.59 | 357.79±179.73 | 3631.57±3623.56 | 122.78±96.38 | 24.31±11.43 | 38.28±32.24 | 431.8±201.99 | 70.26±33.29 | 69.72±31.54 | 2606.06±1109.5 | < 1 time/month | Grilled-food consumption |
| 4406.79±1995.73 | 3644.7±1533.56 | 407.74±189.23 | 4872.81±4355.08 | 148.4±107.35 | 27.65±11.7 | 44.12±37.47 | 484.35±204.41 | 83.83±34.87 | 79.02±31.49 | 2967.53±1128.6 | 1-3 time/month |  |
| 4696.25±2160.26 | 4200.24±1942.45 | 465.95±241.88 | 6153.94±5789.96 | 182.18±168.59 | 31.04±13.83 | 50.48±42.87 | 524.11±218.51 | 94.93±41.69 | 87.46±35.32 | 3249.8±1240.86 | > 3 time/month |  |
| <0.001 | <0.001 | <0.001 | <0.001 | <0.001 | <0.001 | <0.001 | <0.001 | <0.001 | <0.001 | <0.001 | *p* value |  |
| ^I^ Mean ± SD  ^II^ Obtained by One-Way Anova | | | | | | | | | | | | |
